# Aqua(dimethylformamide){tris[(1-methyl-1*H*-benzimidazol-2-yl)methyl]amine}nickel(II) dipicrate

**DOI:** 10.1107/S1600536810029181

**Published:** 2010-07-31

**Authors:** Ke Li, Tao Sun, Yang Yang, Beibei Jia, Huilu Wu

**Affiliations:** a School of Chemical and Biological Engineering, Lanzhou Jiaotong University, Lanzhou 730070, People’s Republic of China

## Abstract

In the title complex, [Ni(C_27_H_27_N_7_)(C_3_H_7_NO)(H_2_O)](C_6_H_2_N_3_O_7_)_2_, the Ni^II^ ion is coordinated in a slightly distorted octa­hedral coordination evironment by an NiN_4_O_2_ ligand set. The tris­(*N*-methyl­benzimidazol-2-ylmeth­yl)amine ligand is in a tetra­dentate mode while a coordinated water molecule and a dimethyl­formamide ligand complete the coordination. In the crystal structure, inter­molecular O—H⋯O hydrogen bonds link the cation and one of the pictrate anions into four-component centrosymmetric clusters.

## Related literature

For the biological activity of compounds containing a benzimid­azole core, see: Horton *et al.* (2003 ▶). For related structures, see Wu *et al.* (2005 ▶, 2009 ▶).
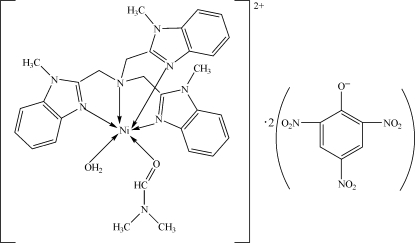


## Experimental

### 

#### Crystal data

[Ni(C_27_H_27_N_7_)(C_3_H_7_NO)(H_2_O)](C_6_H_2_N_3_O_7_)_2_
*M* *_r_* = 1055.57
Triclinic, 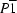

*a* = 12.0768 (3) Å
*b* = 13.2619 (4) Å
*c* = 15.3544 (4) Å
α = 108.583 (1)°
β = 95.703 (1)°
γ = 99.506 (1)°
*V* = 2268.36 (11) Å^3^
*Z* = 2
Mo *K*α radiation
μ = 0.52 mm^−1^
*T* = 153 K
0.25 × 0.22 × 0.11 mm

#### Data collection

Bruker SMART APEXII diffractometer
Absorption correction: multi-scan (*SADABS*; Sheldrick, 1996 ▶) *T*_min_ = 0.882, *T*_max_ = 0.945
18738 measured reflections
8420 independent reflections
5743 reflections with *I* > 2σ(*I*)
*R*_int_ = 0.041

#### Refinement

*R*[*F* ^2^ > 2σ(*F* ^2^)] = 0.050
*wR*(*F* ^2^) = 0.159
*S* = 1.18
8420 reflections
667 parameters
2 restraints
H atoms treated by a mixture of independent and constrained refinement
Δρ_max_ = 1.01 e Å^−3^
Δρ_min_ = −1.24 e Å^−3^

### 

Data collection: *APEX2* (Bruker, 2000 ▶); cell refinement: *SAINT* (Bruker, 2000 ▶); data reduction: *SAINT*; program(s) used to solve structure: *SHELXS97* (Sheldrick, 2008 ▶); program(s) used to refine structure: *SHELXL97* (Sheldrick, 2008 ▶); molecular graphics: *SHELXTL* (Sheldrick, 2008 ▶) and *PLATON* (Spek, 2009 ▶); software used to prepare material for publication: *SHELXTL*.

## Supplementary Material

Crystal structure: contains datablocks I. DOI: 10.1107/S1600536810029181/lh5085sup1.cif

Structure factors: contains datablocks I. DOI: 10.1107/S1600536810029181/lh5085Isup2.hkl

Additional supplementary materials:  crystallographic information; 3D view; checkCIF report

## Figures and Tables

**Table 1:** Hydrogen-bond geometry (Å, °)

*D*—H⋯*A*	*D*—H	H⋯*A*	*D*⋯*A*	*D*—H⋯*A*
O2—H2*D*⋯O3	0.86 (2)	1.86 (2)	2.708 (4)	169 (6)
O2—H2*C*⋯O3^i^	0.85 (2)	1.95 (3)	2.763 (4)	160 (7)

Symmetry code: (i) 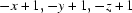.

**Fig. 1.**
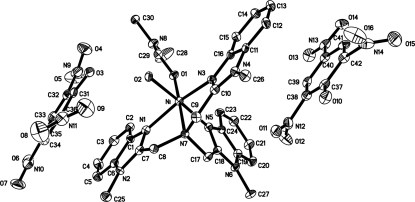
The asymmetric unit of the title compound. Hydrogen atoms have been omitted for clarity and the displacement ellipsoids are shown at the 30% probability level.

**Fig. 2.**
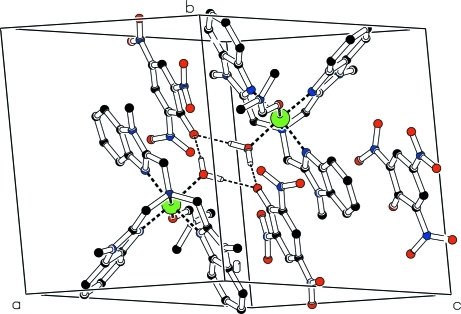
Part of the crystal structure of the title compound, showing hydrogen bonds as dashed lines. Only H atoms involved in hydrogen bonging are shown.

## supplementary crystallographic information

### Comment

We are interested in tris(2-benzimidazolyl)alkanes and their derivatives and we have previously reported the crystal structure of some related complexes (Wu *et al.*, 2009; Wu *et al.*, 2005). The benzimidazole core is of a wide interest because of its diverse biological activities, and it is a well known  structure in medicinal chemistry (Horton *et al.* 2003). The asymmetric unit of the title compound consists of an [Ni(Mentb)(DMF)(H_2_O)] cation (Mentb = tris(*N*-methylbenzimidazol-2-ylmethyl)amine and two picrate anions (Fig.1). The Ni^II^ ion is coordinated in a slightly- distorted octahedral coordination evironment by  NiN_4_O_2_ ligand set. The Mentb ligand coordinates in a tetradentate mode while a coordinated water and a dimethylformamide ligand complete the coordination. In the crystal structure, intermolecular O-H···O hydrogen bonds link the cation and one of the pictrate anions into four component centrosymmetric clusters (Fig. 2).

### Experimental

To a stirred solution of tris(*N*-methylbenzimidazol-2-ylmethyl)amine (0.0899 g, 0.2 mmol) in hot MeOH (10 ml) was added Ni(C_6_H_2_N_3_O_7_)_2_ (0.0103 g, 0.2 mmol) in MeOH (5 ml). A pale green crystalline product formed rapidly. The precipitate was filtered off, washed with MeOH and absolute Et_2_O, and dried *in vacuo*. The dried precipitate was dissolved in DMF to form a pale green solution that was allowed to evaporate at room temperature. Green crystals suitable for X-ray diffraction studies were obtained after three weeks (Yield, 62%). Elemental analysis found: C, 47.79%; H, 3.82%; N, 18.58%; calcd. for C42 H40 N14 O16 Ni: C, 47.82%; H, 3.79%; N, 18.51%.

### Refinement

H atoms bonded to C atoms were placed in calculated positions and included in a riding-model approximation with C—H distances ranging from 0.95 to 0.99 Å and *U*_iso_(H) = 1.2 *U*_eq_(C) or *U*_iso_(H) = 1.5 *U*_eq_(C_methyl_). The water H atoms were located in a difference Fourier map and refined with the restraint O-H = 0.85 (2)Å.

### Crystal data

**Table:**

[Ni(C_27_H_27_N_7_)(C_3_H_7_NO)(H_2_O)](C_6_H_2_N_3_O_7_)_2_	*Z* = 2
*M**_r_* = 1055.57	*F*(000) = 1092
Triclinic, *P*1	*D*_x_ = 1.545 Mg m^−^^3^
Hall symbol: -P 1	Mo *K*α radiation, λ = 0.71073 Å
*a* = 12.0768 (3) Å	Cell parameters from 8420 reflections
*b* = 13.2619 (4) Å	θ = 2.4–28.2°
*c* = 15.3544 (4) Å	µ = 0.52 mm^−^^1^
α = 108.583 (1)°	*T* = 153 K
β = 95.703 (1)°	Block, green
γ = 99.506 (1)°	0.25 × 0.22 × 0.11 mm
*V* = 2268.36 (11) Å^3^	

### Data collection

**Table:**

Bruker SMART APEXII diffractometer	8420 independent reflections
Radiation source: fine-focus sealed tube	5743 reflections with *I* > 2σ(*I*)
graphite	*R*_int_ = 0.041
ω scans	θ_max_ = 25.5°, θ_min_ = 3.1°
Absorption correction: multi-scan (*SADABS*; Sheldrick, 1996)	*h* = −14→14
*T*_min_ = 0.882, *T*_max_ = 0.945	*k* = −16→16
18738 measured reflections	*l* = −17→18

### Refinement

**Table:**

Refinement on *F*^2^	Secondary atom site location: difference Fourier map
Least-squares matrix: full	Hydrogen site location: inferred from neighbouring sites
*R*[*F*^2^ > 2σ(*F*^2^)] = 0.050	H atoms treated by a mixture of independent and constrained refinement
*wR*(*F*^2^) = 0.159	*w* = 1/[σ^2^(*F*_o_^2^) + (0.0452*P*)^2^ + 5.4011*P*] where *P* = (*F*_o_^2^ + 2*F*_c_^2^)/3
*S* = 1.18	(Δ/σ)_max_ = 0.001
8420 reflections	Δρ_max_ = 1.01 e Å^−^^3^
667 parameters	Δρ_min_ = −1.24 e Å^−^^3^
2 restraints	Extinction correction: *SHELXL*, Fc^*^=kFc[1+0.001xFc^2^λ^3^/sin(2θ)]^-1/4^
Primary atom site location: structure-invariant direct methods	Extinction coefficient: 0.0026 (5)

### Special details

**Table:**

Geometry. All e.s.d.'s (except the e.s.d. in the dihedral angle between two l.s. planes) are estimated using the full covariance matrix. The cell e.s.d.'s are taken into account individually in the estimation of e.s.d.'s in distances, angles and torsion angles; correlations between e.s.d.'s in cell parameters are only used when they are defined by crystal symmetry. An approximate (isotropic) treatment of cell e.s.d.'s is used for estimating e.s.d.'s involving l.s. planes.
Refinement. Refinement of *F*^2^ against ALL reflections. The weighted *R*-factor *wR* and goodness of fit *S* are based on *F*^2^, conventional *R*-factors *R* are based on *F*, with *F* set to zero for negative *F*^2^. The threshold expression of *F*^2^ > σ(*F*^2^) is used only for calculating *R*-factors(gt) *etc*. and is not relevant to the choice of reflections for refinement. *R*-factors based on *F*^2^ are statistically about twice as large as those based on *F*, and *R*- factors based on ALL data will be even larger.

### Fractional atomic coordinates and isotropic or equivalent isotropic displacement parameters (Å^2^)

**Table:**

	*x*	*y*	*z*	*U*_iso_*/*U*_eq_	
Ni	0.46074 (4)	0.67214 (4)	0.72572 (4)	0.02153 (16)	
O1	0.3577 (2)	0.7136 (2)	0.63281 (19)	0.0281 (7)	
O2	0.5213 (3)	0.5564 (3)	0.6231 (2)	0.0274 (7)	
O3	0.6467 (3)	0.6084 (3)	0.5008 (2)	0.0476 (10)	
O4	0.5367 (3)	0.6964 (4)	0.3971 (3)	0.0792 (14)	
O5	0.5862 (5)	0.8686 (5)	0.4310 (4)	0.108 (2)	
O6	0.9546 (4)	1.0643 (3)	0.5816 (3)	0.0661 (13)	
O7	1.0726 (4)	0.9877 (4)	0.6429 (3)	0.0731 (14)	
O8	0.9680 (4)	0.6105 (4)	0.5949 (4)	0.0919 (17)	
O9	0.7967 (4)	0.5556 (4)	0.6107 (4)	0.0843 (15)	
O10	0.2350 (3)	0.3427 (3)	0.9903 (2)	0.0412 (8)	
O11	0.3778 (3)	0.5213 (3)	0.9850 (2)	0.0486 (10)	
O12	0.2960 (3)	0.6577 (4)	1.0118 (3)	0.0670 (12)	
O13	−0.0696 (3)	0.6005 (3)	0.8309 (3)	0.0503 (9)	
O14	−0.1952 (3)	0.4559 (3)	0.8155 (2)	0.0456 (9)	
O15	−0.0703 (4)	0.2009 (4)	0.9669 (3)	0.0758 (14)	
O16	0.0677 (4)	0.1511 (3)	0.8988 (4)	0.0825 (16)	
N1	0.6052 (3)	0.7868 (3)	0.7367 (2)	0.0237 (7)	
N2	0.7876 (3)	0.8539 (3)	0.7984 (2)	0.0234 (8)	
N3	0.3619 (3)	0.5380 (3)	0.7390 (2)	0.0237 (8)	
N4	0.3585 (3)	0.3892 (3)	0.7762 (2)	0.0248 (8)	
N5	0.4158 (3)	0.7756 (3)	0.8396 (2)	0.0237 (8)	
N6	0.4450 (3)	0.8522 (3)	0.9935 (2)	0.0251 (8)	
N7	0.5741 (3)	0.6426 (3)	0.8305 (2)	0.0206 (7)	
N8	0.2926 (3)	0.7234 (3)	0.4929 (2)	0.0321 (9)	
N9	0.6035 (4)	0.7845 (5)	0.4381 (3)	0.0539 (13)	
N10	0.9804 (4)	0.9860 (4)	0.5993 (3)	0.0495 (12)	
N11	0.8678 (4)	0.6177 (4)	0.5921 (3)	0.0481 (11)	
N12	0.2919 (3)	0.5600 (4)	0.9830 (3)	0.0397 (10)	
N13	−0.0966 (3)	0.5105 (3)	0.8384 (3)	0.0349 (9)	
N14	0.0128 (4)	0.2193 (4)	0.9307 (3)	0.0484 (12)	
C1	0.6383 (3)	0.8840 (3)	0.7211 (3)	0.0231 (9)	
C2	0.5753 (4)	0.9402 (3)	0.6782 (3)	0.0281 (10)	
H2B	0.4972	0.9119	0.6520	0.034*	
C3	0.6314 (4)	1.0377 (4)	0.6758 (3)	0.0357 (11)	
H3A	0.5911	1.0781	0.6477	0.043*	
C4	0.7468 (4)	1.0795 (4)	0.7138 (4)	0.0380 (11)	
H4A	0.7826	1.1472	0.7103	0.046*	
C5	0.8102 (4)	1.0252 (4)	0.7563 (3)	0.0339 (11)	
H5A	0.8883	1.0535	0.7822	0.041*	
C6	0.7524 (3)	0.9264 (3)	0.7589 (3)	0.0255 (9)	
C7	0.6969 (3)	0.7732 (3)	0.7840 (3)	0.0232 (9)	
C8	0.6925 (3)	0.6766 (3)	0.8150 (3)	0.0250 (9)	
H8A	0.7136	0.6163	0.7667	0.030*	
H8B	0.7468	0.6956	0.8733	0.030*	
C9	0.5451 (3)	0.5244 (3)	0.8168 (3)	0.0262 (9)	
H9A	0.5623	0.5127	0.8768	0.031*	
H9B	0.5903	0.4844	0.7727	0.031*	
C10	0.4205 (3)	0.4843 (3)	0.7788 (3)	0.0248 (9)	
C11	0.2494 (3)	0.3800 (3)	0.7303 (3)	0.0230 (9)	
C12	0.1523 (4)	0.2984 (4)	0.7074 (3)	0.0305 (10)	
H12A	0.1515	0.2337	0.7218	0.037*	
C13	0.0562 (4)	0.3171 (4)	0.6622 (3)	0.0318 (10)	
H13A	−0.0127	0.2640	0.6453	0.038*	
C14	0.0588 (4)	0.4132 (4)	0.6410 (3)	0.0318 (10)	
H14A	−0.0084	0.4232	0.6100	0.038*	
C15	0.1555 (3)	0.4924 (3)	0.6637 (3)	0.0265 (9)	
H15A	0.1565	0.5572	0.6496	0.032*	
C16	0.2530 (3)	0.4742 (3)	0.7088 (3)	0.0232 (9)	
C17	0.5556 (4)	0.7092 (4)	0.9249 (3)	0.0279 (10)	
H17A	0.5276	0.6601	0.9585	0.033*	
H17B	0.6291	0.7560	0.9610	0.033*	
C18	0.4724 (3)	0.7786 (3)	0.9191 (3)	0.0221 (9)	
C19	0.3643 (3)	0.8988 (3)	0.9595 (3)	0.0263 (9)	
C20	0.3085 (4)	0.9796 (4)	1.0048 (3)	0.0350 (11)	
H20A	0.3210	1.0117	1.0707	0.042*	
C21	0.2339 (4)	1.0103 (4)	0.9487 (3)	0.0344 (11)	
H21A	0.1947	1.0658	0.9765	0.041*	
C22	0.2151 (4)	0.9617 (4)	0.8520 (3)	0.0360 (11)	
H22A	0.1627	0.9845	0.8158	0.043*	
C23	0.2704 (3)	0.8811 (4)	0.8072 (3)	0.0313 (10)	
H23A	0.2569	0.8482	0.7413	0.038*	
C24	0.3464 (3)	0.8508 (3)	0.8632 (3)	0.0233 (9)	
C25	0.9037 (4)	0.8644 (4)	0.8443 (3)	0.0360 (11)	
H25A	0.9067	0.8029	0.8661	0.054*	
H25B	0.9253	0.9326	0.8975	0.054*	
H25C	0.9567	0.8648	0.7999	0.054*	
C26	0.3955 (4)	0.3098 (4)	0.8132 (3)	0.0334 (10)	
H26A	0.4758	0.3358	0.8419	0.050*	
H26B	0.3863	0.2402	0.7625	0.050*	
H26C	0.3494	0.2999	0.8600	0.050*	
C27	0.4860 (4)	0.8732 (4)	1.0919 (3)	0.0327 (10)	
H27A	0.5429	0.8297	1.0971	0.049*	
H27B	0.4221	0.8534	1.1220	0.049*	
H27C	0.5205	0.9506	1.1226	0.049*	
C28	0.3449 (3)	0.6785 (3)	0.5465 (3)	0.0247 (9)	
H28A	0.3740	0.6161	0.5172	0.030*	
C29	0.2483 (7)	0.8196 (6)	0.5310 (4)	0.088 (3)	
H29A	0.2621	0.8427	0.5992	0.132*	
H29B	0.1662	0.8037	0.5087	0.132*	
H29C	0.2862	0.8778	0.5112	0.132*	
C30	0.2785 (4)	0.6789 (4)	0.3916 (3)	0.0319 (10)	
H30A	0.3109	0.6136	0.3730	0.048*	
H30B	0.3177	0.7333	0.3682	0.048*	
H30C	0.1973	0.6598	0.3656	0.048*	
C31	0.7227 (4)	0.6930 (4)	0.5180 (3)	0.0322 (10)	
C32	0.7101 (4)	0.7865 (4)	0.4924 (3)	0.0310 (10)	
C33	0.7918 (4)	0.8803 (4)	0.5177 (3)	0.0354 (11)	
H33A	0.7775	0.9406	0.5010	0.042*	
C34	0.8956 (4)	0.8854 (4)	0.5681 (3)	0.0311 (10)	
C35	0.9189 (4)	0.7981 (4)	0.5902 (3)	0.0323 (10)	
H35A	0.9920	0.8014	0.6216	0.039*	
C36	0.8360 (4)	0.7063 (4)	0.5667 (3)	0.0302 (10)	
C37	0.1626 (4)	0.3812 (4)	0.9551 (3)	0.0298 (10)	
C38	0.1809 (3)	0.4884 (4)	0.9463 (3)	0.0313 (10)	
C39	0.0991 (4)	0.5297 (4)	0.9091 (3)	0.0294 (10)	
H39A	0.1170	0.6003	0.9048	0.035*	
C40	−0.0101 (3)	0.4675 (4)	0.8779 (3)	0.0275 (10)	
C41	−0.0389 (4)	0.3658 (4)	0.8866 (3)	0.0326 (11)	
H41A	−0.1149	0.3250	0.8678	0.039*	
C42	0.0447 (4)	0.3260 (4)	0.9229 (3)	0.0291 (10)	
H2D	0.569 (4)	0.575 (5)	0.590 (4)	0.07 (2)*	
H2C	0.479 (5)	0.496 (3)	0.590 (4)	0.10 (3)*	

### Atomic displacement parameters (Å^2^)

**Table:**

	*U*^11^	*U*^22^	*U*^33^	*U*^12^	*U*^13^	*U*^23^
Ni	0.0232 (3)	0.0219 (3)	0.0210 (3)	0.0076 (2)	0.0037 (2)	0.0080 (2)
O1	0.0310 (15)	0.0346 (17)	0.0204 (15)	0.0126 (13)	0.0020 (12)	0.0098 (13)
O2	0.0276 (16)	0.0270 (17)	0.0267 (16)	0.0038 (14)	0.0095 (14)	0.0075 (14)
O3	0.054 (2)	0.044 (2)	0.0298 (18)	−0.0176 (18)	0.0120 (16)	0.0046 (16)
O4	0.042 (2)	0.099 (4)	0.079 (3)	0.006 (3)	−0.011 (2)	0.017 (3)
O5	0.125 (5)	0.082 (4)	0.100 (4)	0.054 (4)	−0.051 (3)	0.014 (3)
O6	0.102 (3)	0.024 (2)	0.072 (3)	−0.002 (2)	0.042 (3)	0.0137 (19)
O7	0.061 (3)	0.074 (3)	0.059 (3)	−0.031 (2)	−0.011 (2)	0.018 (2)
O8	0.082 (3)	0.098 (4)	0.131 (5)	0.051 (3)	0.016 (3)	0.070 (4)
O9	0.105 (4)	0.055 (3)	0.112 (4)	0.008 (3)	0.020 (3)	0.058 (3)
O10	0.0372 (18)	0.059 (2)	0.0392 (19)	0.0253 (17)	0.0060 (15)	0.0247 (17)
O11	0.0251 (17)	0.081 (3)	0.045 (2)	0.0168 (18)	0.0085 (15)	0.025 (2)
O12	0.043 (2)	0.049 (3)	0.102 (4)	−0.0030 (19)	0.006 (2)	0.025 (2)
O13	0.051 (2)	0.055 (2)	0.059 (2)	0.0289 (19)	0.0080 (18)	0.030 (2)
O14	0.0326 (18)	0.056 (2)	0.043 (2)	0.0185 (17)	−0.0091 (15)	0.0090 (17)
O15	0.099 (4)	0.049 (3)	0.073 (3)	−0.007 (3)	0.019 (3)	0.022 (2)
O16	0.064 (3)	0.041 (2)	0.135 (4)	0.026 (2)	−0.011 (3)	0.019 (3)
N1	0.0284 (18)	0.0211 (18)	0.0252 (18)	0.0109 (15)	0.0071 (15)	0.0093 (15)
N2	0.0169 (16)	0.0262 (19)	0.0242 (18)	0.0012 (14)	0.0002 (14)	0.0071 (15)
N3	0.0206 (16)	0.0245 (19)	0.0245 (18)	0.0066 (14)	0.0024 (14)	0.0058 (15)
N4	0.0297 (18)	0.0213 (18)	0.0274 (19)	0.0073 (15)	0.0054 (15)	0.0125 (15)
N5	0.0228 (17)	0.0254 (19)	0.0249 (18)	0.0103 (15)	0.0053 (14)	0.0080 (15)
N6	0.0285 (18)	0.0266 (19)	0.0189 (17)	0.0102 (15)	0.0052 (14)	0.0033 (15)
N7	0.0196 (16)	0.0240 (18)	0.0198 (17)	0.0060 (14)	0.0024 (13)	0.0091 (14)
N8	0.040 (2)	0.033 (2)	0.0263 (19)	0.0138 (18)	0.0021 (16)	0.0118 (17)
N9	0.051 (3)	0.071 (4)	0.035 (2)	0.027 (3)	0.004 (2)	0.006 (2)
N10	0.067 (3)	0.030 (2)	0.042 (3)	−0.010 (2)	0.019 (2)	0.007 (2)
N11	0.065 (3)	0.036 (3)	0.051 (3)	0.015 (2)	0.011 (2)	0.023 (2)
N12	0.032 (2)	0.052 (3)	0.041 (2)	0.008 (2)	0.0115 (18)	0.022 (2)
N13	0.042 (2)	0.043 (2)	0.027 (2)	0.025 (2)	0.0044 (17)	0.0135 (18)
N14	0.051 (3)	0.035 (3)	0.050 (3)	0.011 (2)	−0.013 (2)	0.007 (2)
C1	0.022 (2)	0.020 (2)	0.026 (2)	0.0019 (17)	0.0052 (17)	0.0065 (17)
C2	0.035 (2)	0.027 (2)	0.026 (2)	0.0087 (19)	0.0065 (19)	0.0117 (19)
C3	0.045 (3)	0.030 (3)	0.037 (3)	0.013 (2)	0.008 (2)	0.015 (2)
C4	0.040 (3)	0.025 (2)	0.051 (3)	0.001 (2)	0.010 (2)	0.019 (2)
C5	0.032 (2)	0.028 (2)	0.039 (3)	0.004 (2)	0.006 (2)	0.008 (2)
C6	0.029 (2)	0.024 (2)	0.023 (2)	0.0092 (18)	0.0070 (18)	0.0060 (18)
C7	0.030 (2)	0.024 (2)	0.021 (2)	0.0123 (18)	0.0068 (17)	0.0095 (17)
C8	0.022 (2)	0.031 (2)	0.025 (2)	0.0084 (18)	0.0023 (17)	0.0122 (18)
C9	0.028 (2)	0.026 (2)	0.032 (2)	0.0118 (18)	0.0046 (18)	0.0161 (19)
C10	0.028 (2)	0.024 (2)	0.025 (2)	0.0130 (18)	0.0053 (17)	0.0083 (18)
C11	0.028 (2)	0.024 (2)	0.021 (2)	0.0064 (17)	0.0108 (17)	0.0094 (17)
C12	0.032 (2)	0.025 (2)	0.038 (3)	0.0050 (19)	0.013 (2)	0.013 (2)
C13	0.029 (2)	0.029 (2)	0.040 (3)	0.0050 (19)	0.011 (2)	0.014 (2)
C14	0.024 (2)	0.036 (3)	0.035 (2)	0.0061 (19)	0.0025 (19)	0.012 (2)
C15	0.027 (2)	0.023 (2)	0.033 (2)	0.0069 (18)	0.0065 (18)	0.0112 (19)
C16	0.029 (2)	0.021 (2)	0.023 (2)	0.0065 (17)	0.0099 (17)	0.0100 (17)
C17	0.033 (2)	0.033 (2)	0.020 (2)	0.0121 (19)	0.0049 (18)	0.0085 (18)
C18	0.024 (2)	0.025 (2)	0.021 (2)	0.0069 (17)	0.0024 (16)	0.0121 (17)
C19	0.028 (2)	0.024 (2)	0.029 (2)	0.0096 (18)	0.0071 (18)	0.0091 (18)
C20	0.036 (2)	0.039 (3)	0.028 (2)	0.015 (2)	0.010 (2)	0.004 (2)
C21	0.037 (2)	0.037 (3)	0.034 (3)	0.026 (2)	0.009 (2)	0.009 (2)
C22	0.031 (2)	0.042 (3)	0.039 (3)	0.019 (2)	0.007 (2)	0.014 (2)
C23	0.026 (2)	0.036 (3)	0.028 (2)	0.0089 (19)	0.0006 (18)	0.005 (2)
C24	0.0205 (19)	0.024 (2)	0.024 (2)	0.0066 (17)	0.0040 (16)	0.0046 (17)
C25	0.030 (2)	0.042 (3)	0.036 (3)	0.010 (2)	0.001 (2)	0.014 (2)
C26	0.039 (3)	0.029 (2)	0.037 (3)	0.010 (2)	0.006 (2)	0.017 (2)
C27	0.042 (3)	0.035 (3)	0.017 (2)	0.010 (2)	0.0013 (19)	0.0035 (19)
C28	0.027 (2)	0.023 (2)	0.029 (2)	0.0078 (18)	0.0027 (18)	0.0147 (18)
C29	0.139 (7)	0.097 (6)	0.041 (3)	0.098 (5)	−0.003 (4)	0.010 (3)
C30	0.035 (2)	0.042 (3)	0.021 (2)	0.004 (2)	0.0017 (18)	0.017 (2)
C31	0.036 (2)	0.031 (3)	0.027 (2)	0.002 (2)	0.017 (2)	0.006 (2)
C32	0.032 (2)	0.040 (3)	0.024 (2)	0.017 (2)	0.0084 (18)	0.010 (2)
C33	0.052 (3)	0.033 (3)	0.028 (2)	0.014 (2)	0.014 (2)	0.015 (2)
C34	0.038 (2)	0.021 (2)	0.031 (2)	−0.0034 (19)	0.011 (2)	0.0076 (19)
C35	0.032 (2)	0.034 (3)	0.030 (2)	0.006 (2)	0.0072 (19)	0.009 (2)
C36	0.042 (3)	0.026 (2)	0.029 (2)	0.012 (2)	0.011 (2)	0.0151 (19)
C37	0.033 (2)	0.040 (3)	0.022 (2)	0.021 (2)	0.0073 (18)	0.011 (2)
C38	0.024 (2)	0.045 (3)	0.027 (2)	0.012 (2)	0.0035 (18)	0.013 (2)
C39	0.036 (2)	0.035 (3)	0.024 (2)	0.015 (2)	0.0112 (19)	0.0133 (19)
C40	0.030 (2)	0.036 (3)	0.023 (2)	0.020 (2)	0.0046 (18)	0.0118 (19)
C41	0.035 (2)	0.034 (3)	0.025 (2)	0.015 (2)	0.0026 (19)	0.001 (2)
C42	0.035 (2)	0.029 (2)	0.029 (2)	0.017 (2)	0.0036 (19)	0.0129 (19)

### Geometric parameters (Å, °)

**Table:**

Ni—N5	2.035 (3)	C8—H8A	0.9900
Ni—N3	2.052 (4)	C8—H8B	0.9900
Ni—O1	2.063 (3)	C9—C10	1.500 (6)
Ni—N1	2.071 (3)	C9—H9A	0.9900
Ni—O2	2.106 (3)	C9—H9B	0.9900
Ni—N7	2.181 (3)	C11—C16	1.384 (6)
O1—C28	1.240 (5)	C11—C12	1.388 (6)
O2—H2D	0.86 (2)	C12—C13	1.391 (6)
O2—H2C	0.85 (2)	C12—H12A	0.9500
O3—O3	0.000 (9)	C13—C14	1.409 (6)
O3—C31	1.263 (5)	C13—H13A	0.9500
O4—N9	1.237 (6)	C14—C15	1.366 (6)
O5—N9	1.204 (7)	C14—H14A	0.9500
O6—N10	1.232 (6)	C15—C16	1.401 (6)
O7—N10	1.234 (6)	C15—H15A	0.9500
O8—N11	1.227 (6)	C17—C18	1.485 (6)
O9—N11	1.209 (6)	C17—H17A	0.9900
O10—C37	1.244 (5)	C17—H17B	0.9900
O11—N12	1.233 (5)	C19—C24	1.389 (6)
O12—N12	1.219 (6)	C19—C20	1.394 (6)
O13—N13	1.230 (5)	C20—C21	1.382 (6)
O14—N13	1.239 (5)	C20—H20A	0.9500
O15—N14	1.221 (6)	C21—C22	1.395 (6)
O16—N14	1.216 (6)	C21—H21A	0.9500
N1—C7	1.333 (5)	C22—C23	1.385 (6)
N1—C1	1.387 (5)	C22—H22A	0.9500
N2—C7	1.345 (5)	C23—C24	1.390 (6)
N2—C6	1.388 (5)	C23—H23A	0.9500
N2—C25	1.470 (5)	C25—H25A	0.9800
N3—C10	1.318 (5)	C25—H25B	0.9800
N3—C16	1.387 (5)	C25—H25C	0.9800
N4—C10	1.343 (5)	C26—H26A	0.9800
N4—C11	1.399 (5)	C26—H26B	0.9800
N4—C26	1.455 (5)	C26—H26C	0.9800
N5—C18	1.324 (5)	C27—H27A	0.9800
N5—C24	1.390 (5)	C27—H27B	0.9800
N6—C18	1.355 (5)	C27—H27C	0.9800
N6—C19	1.381 (5)	C28—H28A	0.9500
N6—C27	1.465 (5)	C29—H29A	0.9800
N7—C9	1.490 (5)	C29—H29B	0.9800
N7—C8	1.493 (5)	C29—H29C	0.9800
N7—C17	1.498 (5)	C30—H30A	0.9800
N8—C28	1.329 (5)	C30—H30B	0.9800
N8—C29	1.438 (7)	C30—H30C	0.9800
N8—C30	1.457 (5)	C31—O3	1.263 (5)
N9—C32	1.454 (6)	C31—C32	1.441 (7)
N10—C34	1.450 (6)	C31—C36	1.447 (6)
N11—C36	1.446 (6)	C32—C33	1.372 (7)
N12—C38	1.450 (6)	C33—C34	1.386 (7)
N13—C40	1.441 (5)	C33—H33A	0.9500
N14—C42	1.450 (6)	C34—C35	1.369 (6)
C1—C6	1.388 (6)	C35—C36	1.364 (6)
C1—C2	1.403 (6)	C35—H35A	0.9500
C2—C3	1.371 (6)	C37—C42	1.446 (6)
C2—H2B	0.9500	C37—C38	1.454 (7)
C3—C4	1.403 (7)	C38—C39	1.367 (6)
C3—H3A	0.9500	C39—C40	1.382 (6)
C4—C5	1.383 (7)	C39—H39A	0.9500
C4—H4A	0.9500	C40—C41	1.388 (7)
C5—C6	1.394 (6)	C41—C42	1.363 (6)
C5—H5A	0.9500	C41—H41A	0.9500
C7—C8	1.498 (6)		
			
N5—Ni—N3	92.81 (14)	C14—C13—H13A	119.3
N5—Ni—O1	93.80 (12)	C15—C14—C13	121.8 (4)
N3—Ni—O1	104.09 (12)	C15—C14—H14A	119.1
N5—Ni—N1	90.52 (13)	C13—C14—H14A	119.1
N3—Ni—N1	158.76 (13)	C14—C15—C16	117.4 (4)
O1—Ni—N1	96.60 (13)	C14—C15—H15A	121.3
N5—Ni—O2	170.87 (13)	C16—C15—H15A	121.3
N3—Ni—O2	84.00 (13)	C11—C16—N3	109.2 (4)
O1—Ni—O2	95.28 (12)	C11—C16—C15	120.5 (4)
N1—Ni—O2	89.44 (13)	N3—C16—C15	130.3 (4)
N5—Ni—N7	82.85 (13)	C18—C17—N7	112.0 (3)
N3—Ni—N7	79.47 (12)	C18—C17—H17A	109.2
O1—Ni—N7	175.27 (12)	N7—C17—H17A	109.2
N1—Ni—N7	80.16 (13)	C18—C17—H17B	109.2
O2—Ni—N7	88.15 (12)	N7—C17—H17B	109.2
C28—O1—Ni	128.2 (3)	H17A—C17—H17B	107.9
Ni—O2—H2D	122 (4)	N5—C18—N6	111.8 (3)
Ni—O2—H2C	121 (5)	N5—C18—C17	123.5 (4)
H2D—O2—H2C	108 (6)	N6—C18—C17	124.7 (3)
O3—O3—C31	0(10)	N6—C19—C24	106.3 (3)
C7—N1—C1	105.8 (3)	N6—C19—C20	131.3 (4)
C7—N1—Ni	112.8 (3)	C24—C19—C20	122.3 (4)
C1—N1—Ni	140.8 (3)	C21—C20—C19	116.4 (4)
C7—N2—C6	106.9 (3)	C21—C20—H20A	121.8
C7—N2—C25	127.8 (4)	C19—C20—H20A	121.8
C6—N2—C25	125.3 (4)	C20—C21—C22	121.4 (4)
C10—N3—C16	105.7 (4)	C20—C21—H21A	119.3
C10—N3—Ni	113.2 (3)	C22—C21—H21A	119.3
C16—N3—Ni	140.4 (3)	C23—C22—C21	122.1 (4)
C10—N4—C11	106.6 (3)	C23—C22—H22A	119.0
C10—N4—C26	127.6 (3)	C21—C22—H22A	119.0
C11—N4—C26	125.8 (3)	C22—C23—C24	116.8 (4)
C18—N5—C24	106.2 (3)	C22—C23—H23A	121.6
C18—N5—Ni	113.2 (3)	C24—C23—H23A	121.6
C24—N5—Ni	140.6 (3)	C19—C24—C23	121.0 (4)
C18—N6—C19	107.2 (3)	C19—C24—N5	108.5 (3)
C18—N6—C27	126.9 (4)	C23—C24—N5	130.5 (4)
C19—N6—C27	125.8 (3)	N2—C25—H25A	109.5
C9—N7—C8	111.9 (3)	N2—C25—H25B	109.5
C9—N7—C17	110.7 (3)	H25A—C25—H25B	109.5
C8—N7—C17	111.3 (3)	N2—C25—H25C	109.5
C9—N7—Ni	107.7 (2)	H25A—C25—H25C	109.5
C8—N7—Ni	106.6 (2)	H25B—C25—H25C	109.5
C17—N7—Ni	108.4 (2)	N4—C26—H26A	109.5
C28—N8—C29	122.1 (4)	N4—C26—H26B	109.5
C28—N8—C30	121.8 (4)	H26A—C26—H26B	109.5
C29—N8—C30	116.0 (4)	N4—C26—H26C	109.5
O5—N9—O4	122.3 (5)	H26A—C26—H26C	109.5
O5—N9—C32	118.3 (5)	H26B—C26—H26C	109.5
O4—N9—C32	119.3 (5)	N6—C27—H27A	109.5
O6—N10—O7	124.6 (5)	N6—C27—H27B	109.5
O6—N10—C34	117.6 (5)	H27A—C27—H27B	109.5
O7—N10—C34	117.7 (5)	N6—C27—H27C	109.5
O9—N11—O8	122.9 (5)	H27A—C27—H27C	109.5
O9—N11—C36	119.7 (5)	H27B—C27—H27C	109.5
O8—N11—C36	117.4 (5)	O1—C28—N8	123.7 (4)
O12—N12—O11	122.3 (4)	O1—C28—H28A	118.2
O12—N12—C38	117.8 (4)	N8—C28—H28A	118.2
O11—N12—C38	119.9 (4)	N8—C29—H29A	109.5
O13—N13—O14	123.1 (4)	N8—C29—H29B	109.5
O13—N13—C40	118.5 (4)	H29A—C29—H29B	109.5
O14—N13—C40	118.4 (4)	N8—C29—H29C	109.5
O16—N14—O15	122.8 (5)	H29A—C29—H29C	109.5
O16—N14—C42	118.8 (5)	H29B—C29—H29C	109.5
O15—N14—C42	118.4 (5)	N8—C30—H30A	109.5
N1—C1—C6	108.8 (4)	N8—C30—H30B	109.5
N1—C1—C2	130.5 (4)	H30A—C30—H30B	109.5
C6—C1—C2	120.8 (4)	N8—C30—H30C	109.5
C3—C2—C1	117.0 (4)	H30A—C30—H30C	109.5
C3—C2—H2B	121.5	H30B—C30—H30C	109.5
C1—C2—H2B	121.5	O3—C31—O3	0.0 (4)
C2—C3—C4	121.7 (4)	O3—C31—C32	125.8 (4)
C2—C3—H3A	119.1	O3—C31—C32	125.8 (4)
C4—C3—H3A	119.1	O3—C31—C36	122.6 (4)
C5—C4—C3	122.0 (4)	O3—C31—C36	122.6 (4)
C5—C4—H4A	119.0	C32—C31—C36	111.6 (4)
C3—C4—H4A	119.0	C33—C32—C31	124.4 (4)
C4—C5—C6	115.9 (4)	C33—C32—N9	116.6 (5)
C4—C5—H5A	122.0	C31—C32—N9	119.0 (4)
C6—C5—H5A	122.0	C32—C33—C34	118.7 (4)
N2—C6—C1	106.2 (4)	C32—C33—H33A	120.7
N2—C6—C5	131.1 (4)	C34—C33—H33A	120.7
C1—C6—C5	122.6 (4)	C35—C34—C33	121.4 (4)
N1—C7—N2	112.3 (4)	C35—C34—N10	119.4 (4)
N1—C7—C8	120.9 (4)	C33—C34—N10	119.1 (4)
N2—C7—C8	126.8 (3)	C36—C35—C34	119.2 (4)
N7—C8—C7	108.1 (3)	C36—C35—H35A	120.4
N7—C8—H8A	110.1	C34—C35—H35A	120.4
C7—C8—H8A	110.1	C35—C36—N11	115.7 (4)
N7—C8—H8B	110.1	C35—C36—C31	124.5 (4)
C7—C8—H8B	110.1	N11—C36—C31	119.8 (4)
H8A—C8—H8B	108.4	O10—C37—C42	123.3 (4)
N7—C9—C10	107.8 (3)	O10—C37—C38	125.8 (4)
N7—C9—H9A	110.1	C42—C37—C38	110.7 (4)
C10—C9—H9A	110.1	C39—C38—N12	116.6 (4)
N7—C9—H9B	110.1	C39—C38—C37	124.7 (4)
C10—C9—H9B	110.1	N12—C38—C37	118.7 (4)
H9A—C9—H9B	108.5	C38—C39—C40	119.2 (4)
N3—C10—N4	112.9 (4)	C38—C39—H39A	120.4
N3—C10—C9	122.2 (4)	C40—C39—H39A	120.4
N4—C10—C9	124.8 (4)	C39—C40—C41	121.3 (4)
C16—C11—C12	123.0 (4)	C39—C40—N13	119.4 (4)
C16—C11—N4	105.7 (3)	C41—C40—N13	119.3 (4)
C12—C11—N4	131.3 (4)	C42—C41—C40	118.3 (4)
C11—C12—C13	115.9 (4)	C42—C41—H41A	120.8
C11—C12—H12A	122.0	C40—C41—H41A	120.8
C13—C12—H12A	122.0	C41—C42—C37	125.8 (4)
C12—C13—C14	121.4 (4)	C41—C42—N14	117.4 (4)
C12—C13—H13A	119.3	C37—C42—N14	116.9 (4)
			
N5—Ni—O1—C28	179.5 (3)	C12—C11—C16—C15	1.4 (6)
N3—Ni—O1—C28	−86.7 (4)	N4—C11—C16—C15	−178.5 (4)
N1—Ni—O1—C28	88.5 (4)	C10—N3—C16—C11	−1.1 (5)
O2—Ni—O1—C28	−1.6 (4)	Ni—N3—C16—C11	167.7 (3)
N7—Ni—O1—C28	134.9 (14)	C10—N3—C16—C15	178.6 (4)
N5—Ni—N1—C7	94.7 (3)	Ni—N3—C16—C15	−12.5 (7)
N3—Ni—N1—C7	−4.4 (5)	C14—C15—C16—C11	−1.0 (6)
O1—Ni—N1—C7	−171.4 (3)	C14—C15—C16—N3	179.2 (4)
O2—Ni—N1—C7	−76.2 (3)	C9—N7—C17—C18	−121.2 (4)
N7—Ni—N1—C7	12.1 (3)	C8—N7—C17—C18	113.8 (4)
N5—Ni—N1—C1	−74.3 (4)	Ni—N7—C17—C18	−3.2 (4)
N3—Ni—N1—C1	−173.4 (4)	C24—N5—C18—N6	−0.6 (5)
O1—Ni—N1—C1	19.6 (4)	Ni—N5—C18—N6	176.9 (3)
O2—Ni—N1—C1	114.8 (4)	C24—N5—C18—C17	179.4 (4)
N7—Ni—N1—C1	−156.9 (5)	Ni—N5—C18—C17	−3.1 (5)
N5—Ni—N3—C10	−100.0 (3)	C19—N6—C18—N5	0.7 (5)
O1—Ni—N3—C10	165.4 (3)	C27—N6—C18—N5	176.5 (4)
N1—Ni—N3—C10	−1.3 (6)	C19—N6—C18—C17	−179.3 (4)
O2—Ni—N3—C10	71.4 (3)	C27—N6—C18—C17	−3.6 (7)
N7—Ni—N3—C10	−17.8 (3)	N7—C17—C18—N5	4.4 (6)
N5—Ni—N3—C16	91.7 (4)	N7—C17—C18—N6	−175.5 (4)
O1—Ni—N3—C16	−2.9 (4)	C18—N6—C19—C24	−0.5 (5)
N1—Ni—N3—C16	−169.6 (4)	C27—N6—C19—C24	−176.3 (4)
O2—Ni—N3—C16	−96.9 (4)	C18—N6—C19—C20	−178.9 (5)
N7—Ni—N3—C16	173.9 (4)	C27—N6—C19—C20	5.2 (8)
N3—Ni—N5—C18	79.7 (3)	N6—C19—C20—C21	177.9 (5)
O1—Ni—N5—C18	−175.9 (3)	C24—C19—C20—C21	−0.3 (7)
N1—Ni—N5—C18	−79.3 (3)	C19—C20—C21—C22	0.8 (7)
O2—Ni—N5—C18	10.5 (10)	C20—C21—C22—C23	−0.6 (8)
N7—Ni—N5—C18	0.7 (3)	C21—C22—C23—C24	−0.2 (7)
N3—Ni—N5—C24	−104.1 (4)	N6—C19—C24—C23	−179.1 (4)
O1—Ni—N5—C24	0.2 (4)	C20—C19—C24—C23	−0.5 (7)
N1—Ni—N5—C24	96.9 (4)	N6—C19—C24—N5	0.1 (5)
O2—Ni—N5—C24	−173.4 (7)	C20—C19—C24—N5	178.8 (4)
N7—Ni—N5—C24	176.9 (4)	C22—C23—C24—C19	0.7 (7)
N5—Ni—N7—C9	121.3 (3)	C22—C23—C24—N5	−178.3 (4)
N3—Ni—N7—C9	27.1 (2)	C18—N5—C24—C19	0.3 (5)
O1—Ni—N7—C9	166.3 (14)	Ni—N5—C24—C19	−176.0 (3)
N1—Ni—N7—C9	−146.9 (3)	C18—N5—C24—C23	179.4 (5)
O2—Ni—N7—C9	−57.1 (3)	Ni—N5—C24—C23	3.1 (8)
N5—Ni—N7—C8	−118.4 (3)	Ni—O1—C28—N8	−167.6 (3)
N3—Ni—N7—C8	147.3 (3)	C29—N8—C28—O1	1.6 (8)
O1—Ni—N7—C8	−73.5 (15)	C30—N8—C28—O1	−179.4 (4)
N1—Ni—N7—C8	−26.6 (2)	O3—O3—C31—C32	0.00 (4)
O2—Ni—N7—C8	63.1 (2)	O3—O3—C31—C36	0.00 (17)
N5—Ni—N7—C17	1.5 (3)	O3—C31—C32—C33	175.3 (4)
N3—Ni—N7—C17	−92.8 (3)	O3—C31—C32—C33	175.3 (4)
O1—Ni—N7—C17	46.4 (16)	C36—C31—C32—C33	−5.2 (6)
N1—Ni—N7—C17	93.2 (3)	O3—C31—C32—N9	−3.0 (7)
O2—Ni—N7—C17	−177.0 (3)	O3—C31—C32—N9	−3.0 (7)
C7—N1—C1—C6	1.1 (4)	C36—C31—C32—N9	176.5 (4)
Ni—N1—C1—C6	170.6 (3)	O5—N9—C32—C33	−11.2 (7)
C7—N1—C1—C2	−177.1 (4)	O4—N9—C32—C33	165.0 (5)
Ni—N1—C1—C2	−7.6 (8)	O5—N9—C32—C31	167.2 (5)
N1—C1—C2—C3	177.8 (4)	O4—N9—C32—C31	−16.6 (7)
C6—C1—C2—C3	−0.2 (6)	C31—C32—C33—C34	2.7 (7)
C1—C2—C3—C4	0.4 (7)	N9—C32—C33—C34	−178.9 (4)
C2—C3—C4—C5	−0.5 (8)	C32—C33—C34—C35	2.1 (7)
C3—C4—C5—C6	0.2 (7)	C32—C33—C34—N10	−176.9 (4)
C7—N2—C6—C1	−0.2 (4)	O6—N10—C34—C35	−176.9 (4)
C25—N2—C6—C1	178.2 (4)	O7—N10—C34—C35	2.0 (7)
C7—N2—C6—C5	177.5 (4)	O6—N10—C34—C33	2.1 (7)
C25—N2—C6—C5	−4.1 (7)	O7—N10—C34—C33	−179.0 (4)
N1—C1—C6—N2	−0.6 (5)	C33—C34—C35—C36	−3.8 (7)
C2—C1—C6—N2	177.8 (4)	N10—C34—C35—C36	175.2 (4)
N1—C1—C6—C5	−178.5 (4)	C34—C35—C36—N11	179.7 (4)
C2—C1—C6—C5	−0.1 (6)	C34—C35—C36—C31	0.7 (7)
C4—C5—C6—N2	−177.2 (4)	O9—N11—C36—C35	149.3 (5)
C4—C5—C6—C1	0.1 (7)	O8—N11—C36—C35	−29.7 (7)
C1—N1—C7—N2	−1.3 (5)	O9—N11—C36—C31	−31.7 (7)
Ni—N1—C7—N2	−174.1 (3)	O8—N11—C36—C31	149.3 (5)
C1—N1—C7—C8	178.9 (4)	O3—C31—C36—C35	−177.0 (4)
Ni—N1—C7—C8	6.1 (5)	O3—C31—C36—C35	−177.0 (4)
C6—N2—C7—N1	1.0 (5)	C32—C31—C36—C35	3.5 (6)
C25—N2—C7—N1	−177.4 (4)	O3—C31—C36—N11	4.1 (7)
C6—N2—C7—C8	−179.3 (4)	O3—C31—C36—N11	4.1 (7)
C25—N2—C7—C8	2.4 (7)	C32—C31—C36—N11	−175.4 (4)
C9—N7—C8—C7	152.8 (3)	O12—N12—C38—C39	27.7 (6)
C17—N7—C8—C7	−82.8 (4)	O11—N12—C38—C39	−152.9 (4)
Ni—N7—C8—C7	35.2 (4)	O12—N12—C38—C37	−149.4 (5)
N1—C7—C8—N7	−29.5 (5)	O11—N12—C38—C37	29.9 (6)
N2—C7—C8—N7	150.7 (4)	O10—C37—C38—C39	−178.5 (4)
C8—N7—C9—C10	−147.5 (3)	C42—C37—C38—C39	−2.4 (6)
C17—N7—C9—C10	87.8 (4)	O10—C37—C38—N12	−1.6 (7)
Ni—N7—C9—C10	−30.6 (4)	C42—C37—C38—N12	174.5 (4)
C16—N3—C10—N4	0.6 (5)	N12—C38—C39—C40	−176.3 (4)
Ni—N3—C10—N4	−171.7 (3)	C37—C38—C39—C40	0.7 (7)
C16—N3—C10—C9	177.0 (4)	C38—C39—C40—C41	2.3 (6)
Ni—N3—C10—C9	4.7 (5)	C38—C39—C40—N13	−179.8 (4)
C11—N4—C10—N3	0.2 (5)	O13—N13—C40—C39	2.2 (6)
C26—N4—C10—N3	−180.0 (4)	O14—N13—C40—C39	−176.2 (4)
C11—N4—C10—C9	−176.2 (4)	O13—N13—C40—C41	−179.8 (4)
C26—N4—C10—C9	3.7 (7)	O14—N13—C40—C41	1.8 (6)
N7—C9—C10—N3	19.0 (5)	C39—C40—C41—C42	−3.0 (6)
N7—C9—C10—N4	−165.0 (4)	N13—C40—C41—C42	179.0 (4)
C10—N4—C11—C16	−0.9 (4)	C40—C41—C42—C37	1.0 (7)
C26—N4—C11—C16	179.3 (4)	C40—C41—C42—N14	−179.6 (4)
C10—N4—C11—C12	179.2 (4)	O10—C37—C42—C41	177.8 (4)
C26—N4—C11—C12	−0.6 (7)	C38—C37—C42—C41	1.6 (6)
C16—C11—C12—C13	−1.0 (6)	O10—C37—C42—N14	−1.6 (7)
N4—C11—C12—C13	178.9 (4)	C38—C37—C42—N14	−177.8 (4)
C11—C12—C13—C14	0.3 (7)	O16—N14—C42—C41	128.6 (5)
C12—C13—C14—C15	−0.1 (7)	O15—N14—C42—C41	−49.3 (6)
C13—C14—C15—C16	0.4 (7)	O16—N14—C42—C37	−51.9 (6)
C12—C11—C16—N3	−178.8 (4)	O15—N14—C42—C37	130.2 (5)
N4—C11—C16—N3	1.3 (4)		

### Hydrogen-bond geometry (Å, °)

**Table:**

*D*—H···*A*	*D*—H	H···*A*	*D*···*A*	*D*—H···*A*
O2—H2D···O3	0.86 (2)	1.86 (2)	2.708 (4)	169 (6)
O2—H2C···O3^i^	0.85 (2)	1.95 (3)	2.763 (4)	160 (7)

Symmetry codes:  (i) −*x*+1, −*y*+1, −*z*+1.
